# Supplementary material for: CMAC: A deep learning framework for absolute cardiomyocyte transcriptional age estimation
Source: bioRxiv. 2026 Sep 24:2026.09.19.752897. Preprint. [Version 1] doi: 10.64898/2026.09.19.752897 (PMC13622189; doi:10.64898/2026.09.19.752897)
Supplement: Supplementary file 1 [file media-1.pdf]

Dataset	Author	Isolation	Sequencing	Mapping	Datatype	TImepoints	Accession_number	Species	sample_type
chromium_data	10x Chromium Demo	10x Chromium V3	10x Chromium	CellRanger	UMIs	e18	10x website	mouse	in vivo
desoysa_data	De Soysa	10x Chromium V2	10x Chromium	CellRanger	UMIs	e7.75, e8.25, e9.25	GSE126128	mouse	in vivo
dong_data	Dong	Picked	8bp-STRT-seq	Kallisto/Bustools	UMIs	e9.5, e10.5, e11.5	GSE87038	mouse	in vivo
goodyer_avn_data	Goodyer AVN	10x Chromium V2	10x Chromium	CellRanger	UMIs	e16.5	GSE132658	mouse	in vivo
goodyer_pf_data	Goodyer PF	10x Chromium V2	10x Chromium	CellRanger	UMIs	e16.5	GSE132658	mouse	in vivo
hill10_data	Hill e10.5	10x Chromium V2	10x Chromium	CellRanger	UMIs	e10.5	GSE131181	mouse	in vivo
hill13_data	Hill e13.5	10x Chromium V2	10x Chromium	CellRanger	UMIs	e13.5	GSE131181	mouse	in vivo
jhu_p7_ctr	Suh, Chen	Langendorf-free, Ackers-Johnson	Parse split-seq	Trailmaker/split-pipe	UMIs	p7	unpublished	mouse	in vivo
jhu_p14_ctr	Suh, Chen	Langendorf-free, Ackers-Johnson	Parse split-seq	Trailmaker/split-pipe	UMIs	p14	unpublished	mouse	in vivo
kannan_ref_data	Kannan Reference	LP-FACS	mcSCRB-seq	zUMIs	UMIs	e18, e14, p0, p4, p14, p22, p28, p35, p56, p18, p11, p8	GSE147807	mouse	in vivo
li10_data	Li 10x	10x Chromium V2	10x Chromium	CellRanger	UMIs	e10.5	GSE76118	mouse	in vivo
murphy_data	Murphy	LP-FACS	SCRB-seq	zUMIs	UMIs	p0, p7, p28, p21, p14	GSE165917	mouse	in vivo
northwestern_ctr	Suh		Parse split-seq	Trailmaker/split-pipe	UMIs	e12.5, e14.5, e16.5, P0, P7	unpublished	mouse	in vivo
pijuansala_data	Pijuan-Sala, Griffiths, and Guibentif	10x Chromium V1	10x Chromium	CellRanger	UMIs	e7.75, e8.0, e8.5, e8.25	doi:10.1038/s41586-019-0933-9	mouse	in vivo
wang_dev_data	Wang and Yao	iCell8	Unknown	STAR/FeatureCounts	UMIs	p1, p4, p7, p14	GSE122706	mouse	in vivo
wunnemann_data	Wunnemann	Drop-seq	Drop-seq	Drop-seq Tools	UMIs	e14.5, e16.5, e18.5, p1, p4, p7	GSE150817, GSE109247	mouse	in vivo
xiao_data	Xiao	Drop-seq	Drop-seq	Drop-seq Tools	UMIs	e13.5, e14.5	GSE100861	mouse	in vivo
xiong_data	Xiong, Luo, Yue, and Zhang	Picked	8bp-STRT-seq	Kallisto/Bustools	UMIs	e9.25, e8.25, e8.75	GSE108963	mouse	in vivo
zureick_P14	Zureick, Chen	Langendorf-free, Ackers-Johnson	Parse split-seq	Trailmaker/split-pipe	UMIs	p14	unpublished	mouse	in vivo
